# Application of 16S rRNA gene sequencing in *Helicobacter pylori* detection

**DOI:** 10.7717/peerj.9099

**Published:** 2020-05-13

**Authors:** Aleksander Szymczak, Stanisław Ferenc, Joanna Majewska, Paulina Miernikiewicz, Jan Gnus, Wojciech Witkiewicz, Krystyna Dąbrowska

**Affiliations:** 1 Hirszfeld Institute of Immunology and Experimental Therapy, Polish Academy of Sciences, Wrocław, Poland; 2 Regional Specialist Hospital in Wrocław, Research and Development Center, Wrocław, Poland; 3 Medical Academy in Wroclaw, Wrocław, Poland

**Keywords:** *Helicobacter*, NGS, Sequencing, *Pylori*, Stomach, Next-generation sequencing, Biopsy, Specimens

## Abstract

*Helicobacter pylori* is one of the major stomach microbiome components, promoting development of inflammation and gastric cancer in humans. *H. pylori* has a unique ability to transform into a coccoidal form which is difficult to detect by many diagnostic methods, such as urease activity detection, and even histopathological examination. Here we present a comparison of three methods for *H. pylori* identification: histological assessment (with eosin, hematoxylin, and Giemsa staining), polymerase chain reaction (PCR) detection of urease (ureA specific primers), and detection by 16S rRNA gene sequencing. The study employed biopsies from the antral part of the stomach (*N* = 40). All samples were assessed histologically which revealed *H. pylori* in eight patients. Bacterial DNA isolated from the bioptates was used as a template for PCR reaction and 16S rRNA gene sequencing that revealed *H. pylori* in 13 and in 20 patients, respectively. Thus, 16S rRNA gene sequencing was the most sensitive method for detection of *H. pylori* in stomach biopsy samples.

## Introduction

The constantly growing interest in the human microbiome has revealed complicated connections between its particular components and human physiology. Particularly, over- and underrepresentation of individual bacterial families can be a significant factor inducing homeostasis imbalance, with a prominent effect on human health (Maruvada et al., 2017; Luca et al., 2018; Goodman & Gardner, 2018). This effect includes for instance links between microbiome composition and cancer prevalence and development (Bhatt, Redinbo & Bultman, 2017; Goodman & Gardner, 2018; Gopalakrishnan et al., 2018), while the GLOBOCAN database from the International Agency for Research on Cancer identified stomach cancer as the second most often cause of cancer-related death worldwide in the year 2018 (Ferlay et al., 2019). A microbial species existing within the stomach microbiome, *Helicobacter pylori* is particularly relevant for the occurrence of stomach cancer disease. Probably the first observation of a pathogen contributing to gastric cancer development was performed by a Polish researcher from Jagiellonian University of Cracow as early as in the 19th century. In 1886 Professor W. Jaworski identified a spiral bacterium and named it “*Vibrio rogula”*. He concluded that this bacterium could be an etiological factor of gastrointestinal diseases (Konturek, 2003). However, due to extremely low pH and low access to oxygen, the stomach seemed to be impossible for inhabitation even for microbes. As a result, it was generally considered sterile until the observations in 1984 by Marshall and Warren, who provided evidence for the existence of a spiral pathogen in the antral part of the stomach, and linked its presence to gastritis and ulcers. The bacterium was initially named *Campylobacter pyloridis;* after examination of its genome this bacterium was finally assigned to the genus *Helicobacter* (Marshall & Warren, 1984; Goodwin, 1994). Currently, *Helicobacter pylori* is known to be one of the major factors promoting inflammation and gastric cancer development in humans (Wroblewski & Peek, 2016; Ferenc et al., 2017).

This bacterium demonstrates several adaptations for the difficult environment of stomach. A major factor that allows *H. pylori* to survive in the acidic environment of the stomach is its capability to secrete the enzyme urease. This multimeric enzyme consists of several heterodimers and it catalyses metabolism of urea to CO_2_ and NH_3_, thus locally neutralizing acidity, and creating “a buffer layer” around *H. pylori* cells. Intracellular production of urease in *Helicobacter* can be as high as 10–15% of all proteins expressed in the cell. Notably, urease-negative *H. pylori* mutants are characterized by decreased pathogenicity (Tsuda et al., 1994; Bauerfeind et al., 1997; Kavermann et al., 2003; Debowski et al., 2017). *Helicobacter pylori* also has flagella on the cellular surface, which allow for bacterial movement and better adhesion to gastric epithelial cells (Bhatt, Redinbo & Bultman, 2017). This bacterium passes through the gastric mucus which covers the outer layer of stomach cells, due to chemotaxis receptor genes whose expression provides pH-based coordination (Aihara et al., 2014).

*H. pylori* has an ability to transform into coccoidal form. This transformation can be induced by detrimental environmental conditions such as variable pH, occurrence of effective antibiotics, and increased oxygen exposure. In this case the bacterial cell remains enzymatically inactive and can be defined as viable but non-culturable. This causes difficulties in both *H. pylori* detection and in treatment. Bacterial cells in coccoidal form can survive prolonged exposition to antibiotics and they can be efficiently transmitted between individuals or they can cause recurrent infections (Faghri et al., 2014; Mazaheri Assadi et al., 2015; Poursina et al., 2018). Moreover, *H. pylori* can be resistant to various antibiotics and thus anti-*Helicobacter* therapy often needs to combine two or more chemotherapeutics (Wang et al., 2017). These strains may also have reduced enzymatic activity and wider tolerance for environment pH which makes them more difficult to detect by commonly used enzymatic kits. Additionally, bacteria that transform to coccoidal forms do not loose virulence factors and they are fully capable to turn into aggressive forms after treatment. Recent studies suggest that coccoid *H. pylori* plays crucial role in development of active gastritis in human stomach. This creates a need for testing based on other factors than bacterial metabolites (Tominaga et al., 1999; Reshetnyak & Reshetnyak, 2017; Syahniar et al., 2019).

Most of the negative symptoms in *Helicobacter-* infected patients are caused by VacA (Vacuolating cytotoxin) and CagA (cytotoxin-associated antigen A) proteins. These factors can lead to characteristic vacuolisation in epithelial cells and activation of apoptosis. VacA destabilises homeostasis of human cells through interference with metabolic pathways. *H. pylori* genotype linked with possibility of VacA secretion is strictly associated with the ability of apoptosis induction in gastric epithelial cells. This toxin was classified as a pore forming protein. Though its enzymatic activity was not confirmed, VacA enters host cells. First, it binds to the cells surface and increases permeability of plasma membrane by depolarisation. *In vitro* research demonstrated that VacA induced vacualation of host cells, autophagy, disruption of mitochondrial functions, interruption of cell signalling, and alteration of function in many types of immune cells (such as lymphocytes and macrophages). It also inhibits gastric acid secretion via parietal cells disruption. Intracellular trafficking of VacA is still not well described. The *vacA* gene occurs in all *H. pylori* isolates, however it may occur as different alleles that define an isolate pathogenicity. The key differences were found in the signal region (*s*) and the middle region (*m*), since s1/m1 type produces high amount of toxin, while s2/m2 carriers nearly do not synthesize VacA (Wu et al., 2014; Kim et al., 2015; Foegeding et al., 2016; Bakhti, Latifi-Navid & Zahri, 2020). *CagA* is a marker gene of *cag*-PAI pathogenicity island in a *H. pylori* isolate. Ability to produce CagA protein by an infecting strain of *Helicobacter* is strongly related to increased risk of gastric cancer in an infected patient (Hashi et al., 2018). It has been documented that CagA affects cell proliferation and differentiation cycles, which can lead to gastric carcinoma development. CagA is introduced into gastric epithelial cells through the bacterial type IV secretion system. Recent studies showed that carcinoembryonic antigen-related cell adhesion molecules (CEACAMs) are crucial for CagA delivery. This toxin binds to the surface of the host cell membrane and then process of the uptake occurs, however its mechanism has not been described in details. Oncogenic activity of CagA is mediated by interactions with SHP-2 (tyrosine phosphatase), by interrupting host signalling factors and increasing protooncogenic pathways (Hatakeyama, 2004; Hatakeyama, 2008; Stein et al., 2013; Vaziri et al., 2015). CagA and VacA, but also others, e.g., BabA, SabA and OipA, have been reported as involved in carcinogenesis and inflammatory responses in host cells (Ahmad et al., 2009; Hanada et al., 2014; Zhang et al., 2016; Königer et al., 2017).

Methods for *H. pylori* detection are crucial for preventing and counteracting negative effects of these bacteria. They depend on the type of available sample and on specific characteristics of possible symptoms in a patient. Both invasive and non-invasive diagnostics can be used, and they differ in their specificity and sensitivity. One of the most common is the urease breath test (UBT) that makes use of urease activity in gastric mucosa as a marker of *H. pylori* infection. Patients are supplemented with labelled isotopes C^13^ or C^14^, that eventually allow for the detection: active urease carries isotopes to produced carbon dioxide, so the presence of the isotopes can be measured in the exhaled air after a specific amount of time. Meta-analyses showed that detection methods based on C^13^ allowed for nearly 96% sensitivity and 94% specificity of detection, and C^14^-based methods allowed for 97% and 91%, respectively, where histology and rapid urease tests (direct pH testing in bioptates) were used as the gold standards. The important drawbacks of the UBT method are its relatively high cost and the long (3 h) measurement required (Ferwana et al., 2015). A popular test based on urease activity is pH measurement. Commonly called Rapid Urease Test (RUT), it allows to detect metabolic activity of *H. pylori* in samples. In a sampled tissue, urease activity is measured by pH testing in detection buffers. However, this kind of diagnostics requires an endoscopy procedure with a biopsy of the gastric endothelium. The method shows up to 82% of sensitivity and 90% of specificity. The main disadvantage is that RUT seems to decrease its relibility in case of proton pomps inhibitors use (Adu-Aryee et al., 2016; McNicholl et al., 2017; Dahlén et al., 2018).

Biopsy can also be used for identification of *H. pylori* in microbiological cultures. Due to the high sensitivity of *H. pylori* to external environment conditions, samples must be immediately processed to cultures. This method is the most time consuming, but it can be used to determine *H. pylori* antibiotic resistance. Accuracy of detection by microbial culturing can be strongly affected by the level of practical experience of laboratory staff and it is a time consuming method with results delayed at least 48 h (Parsonnet, Shmuely & Haggerty, 1999; Ndip et al., 2003; Patel et al., 2014b).

Bioptates are commonly used for histological diagnostics of *H. pylori.* Samples are processed by Giemsa stain for bacteria identification followed by haematoxylin and eosin for assessment of inflammatory processes by individual microscopic observation (Chan et al., 1994; Patel et al., 2014b; Sabbagh et al., 2019). Unified scheme of gastritis scoring: Sydney system was first introduced in 1990. It was designed as a set of all-purpose rules to assess gastritis in patients around the world, including those related to *H. pylori* infections, making use of parameters observed during histological examination. In the Sydney system, gastritis should be classified according to an aetiology of a disease (*prefix*), topography (*core*), and morphology (*suffix*). Morphology includes assessment of inflammation, activity (of immune cells), atrophy, intestinal metaplasia, and *H. pylori* presence. The system was updated in 1997 by covering also specimens from angulus of the stomach (Langner et al., 2009; Siddique et al., 2014).

Serological methods for *H. pylori* detection can also be used. Serum, saliva, and even urine are appropriate to use *Helicobacter*-specific IgG testing. Infection with *H. pylori* can be detected indirectly by enzyme-linked immunosorbent assay (ELISA) and enzyme immunoassay (EIA) methods. However serological tests are limited by long delays in possible detection: anti-*Helicobacter* antibodies can be detected only when the immune response has had enough time to develop, and, on the other hand, they can be detected even a long time after *H. pylori* eradication. Once induced during infection, antibodies specific to *H. pylori* can be detected in patients’ sera for weeks (Vaira et al., 1999; Ueda et al., 2014; Sabbagh et al., 2019). This can lead to both false negative and false positive results.

Novel DNA-based techniques are also recommended for *H. pylori* detection. Polymerase chain reaction (PCR) is commonly used and it demonstrates up to 95% sensitivity and 95% specificity. This kind of identification can be done in bioptates but also in stool samples, thus PCR-based detection can be considered either an invasive or non-invasive method of *H. pylori* depending on the applied material. Importantly, DNA-based methods allow for detection of not only active *H. pylori,* it gives a positive response also in case of coccoid forms that may result from stress conditions, e.g., induced by antibiotics, making urease-based detection methods ineffective (He et al., 2002; Momtaz et al., 2012; Sabbagh et al., 2019).

In spite of the recent burst of DNA-based technologies in novel diagnostics, other techniques (than PCR) have not been employed in *H. pylori* detection yet. One of potential approaches is the microbiome analysis by NGS sequencing that targets bacterial 16S rRNA genes, revealing potential presence of a specific bacterial taxon even from trace amounts of samples. However, applicability, sensitivity, and specificity of 16S rRNA gene sequencing for *H. pylori* detection has never been demonstrated so far. Here we present a comparison of three methods for *H. pylori* identification: (i) microscopical identification of Giemsa-stained *H. pylori* together with Sydney scoring of histological samples, (ii) *ureA* gene detection by polymerase chain reaction (PCR), (iii) 16S rRNA genes sequencing by the next-generation sequencing (NGS) methods. This is the first comparison that includes the commonly used detection methods (histology, PCR) and NGS-based metagenomics with 16S rRNA genes sequencing to identify *H. pylori* in patients, and their assessment as potential diagnostic indicators.

## Materials and Methods

### Biopsy from antral part of the stomach

Biopsies from the antral part of the stomach were collected in the Endoscopy Laboratory of the Regional Specialist Hospital in Wroclaw (Poland) by an expert physician from adult patients with their written consent. Patients were recruited as qualified for gastroscopic examination due to adverse symptoms from the GI tract, but without any further specifications, thus they were selected randomly in terms of presence/lack *H. pylori* infection. The exclusion criteria were: antibiotic treatment in last six months, and diagnosed development of stomach cancer. Antral derived biopsies were collected according to hospital procedures for surgeons, based on Updated Sydney System recommendations. Forty samples were assigned to the study. Collection of all types of samples as well as the interview (for the antibiotic use) before examination has been approved by the local Commission of Bioethics, Regional Specialist Hospital Wroclaw, no KB/nr8/rok2017. Parallel to the NGS analysis, histological diagnostics of all samples was conducted in the hospital specialized laboratory according to the updated Sydney system: eosin and haematoxylin stain and eventual scoring (0–3) of possible metaplasia, inflammation, atrophy, and polymorph activity. *H. pylori* was directly identified by microscopic assessment with Giemsa stain. Material for further microbial DNA isolation was immediately placed in 2 ml of sterile phosphate buffered saline (PBS). Our Study included 21 samples derived from women in age from 18 to 81 and 19 specimens from men in age from 30 to 78.

### Bacterial DNA isolation

Each sample was incubated in 2 ml of PBS for 1 h with gentle shaking and next centrifuged for 10 min at 10,000 RPM at 4 °C. The pellet was used for bacterial DNA isolation: it was homogenized and processed with a Bead Beat gravity kit (A&A Biotechnology), which provides optimised conditions for acquisition of bacterial genetic material including *H. pylori* coccoid forms. Initial DNA quality assessment and quantification was done with a Thermo Scientific NanoDrop 2000c spectrophotometer. Isolated samples received unique IDs and were stored in Regional Specialist Hospital DNA Biobank.

### Detection of *Helicobacter pylori* 16S rRNA (next generation sequencing)

Quantitation of isolated DNA was completed by a Qubit 2.0 fluorometer with the Qubit HS DNA Assay Kit. The 16S rRNA library was prepared with the Ion 16S Metagenomics Kit (Thermo Fisher Scientific), according to the manufacturer’s instructions (2 ng of isolated bacterial DNA was used). Kit used in this experiment contains primers covering V2, V4, V8, V3-6, V7-9 hypervariable regions of 16S rRNA gene (sequences of the primers have not been revealed by the manufacturer ThermoFisher Scientific). Barcoding was done with IonXpress Barcode Adapters (Thermo Fisher Scientific). Samples were purified with the Agencourt AMPure XP Kit (Beckman Coulter). Final library quantitation was performed by qPCR with the Ion Library TaqMan Quantitation Kit (Thermo Fisher Scientific) and StepOnePlus Software. The direct sequencing of amplified DNA from the 16S rRNA was conducted in the Ion PGM System for Next-Generation Sequencing (Thermo Fisher Scientific). Briefly, the Ion OneTouch 2 system with Ion PGM Hi-Q View OT2 Kits (Thermo Fisher Scientific) were used for emulsion PCR and enrichment. Sequencing was done with Ion 316 Chips. Data analysis was performed with IonReporter version 5.6 (Thermo Fisher Scientific) and MG-RAST server pipeline. Reads analysed in this experiment are available online (MG-RAST ID: mgp86818) (Keegan, Glass & Meyer, 2016). A minimum of 10 reads in both tools with 99% identity of the 16S rRNA gene was accepted as a positive result, where 99% was the cut-of. Reads were mapped with the MicroSEQ ID and GreenGenes 16S rRNA databases.

### Detection of *Helicobacter pylori* by PCR

The same bacterial DNA samples (as used for NGS reaction) was further used as a template for PCR reaction to identify *H. pylori*. For this purpose an established method with PCR targeting *ureA* gene primers was used: forward –5′-GAGAATGAGATGAAACTCACCC-3′, reverse –5′-GAGAATGAGATGAAACTCACCC-3′ (He et al., 2002; Patel et al., 2014a; Samareh-Fekri et al., 2016). PCR reaction was conducted with TaqMan Universal PCR Master Mix (Thermo Fisher Scientific) according to He et al. (2002): initial denaturation: 95 °C for 10 min, 35 cycles of: denaturation in 94 °C for 30 s, primer annealing at 54 °C for 1 min, product elongation at 72 °C for 1 min. The reaction was completed by final elongation at 72 °C for 10 min. Products were analysed by electrophoresis in 2% agarose with addition of EtBr and 100 ms UV exposure (He et al., 2002). Results were further verified by additional analysis of UV spectrum was performed using Fiji software (Schindelin et al., 2012)**.**

For statistical evaluation McNemar’s test was used in the R environment, as previously described (Peng et al., 2009; Moon et al., 2012; Miftahussurur et al., 2017).

## Results

**Figure 1:**
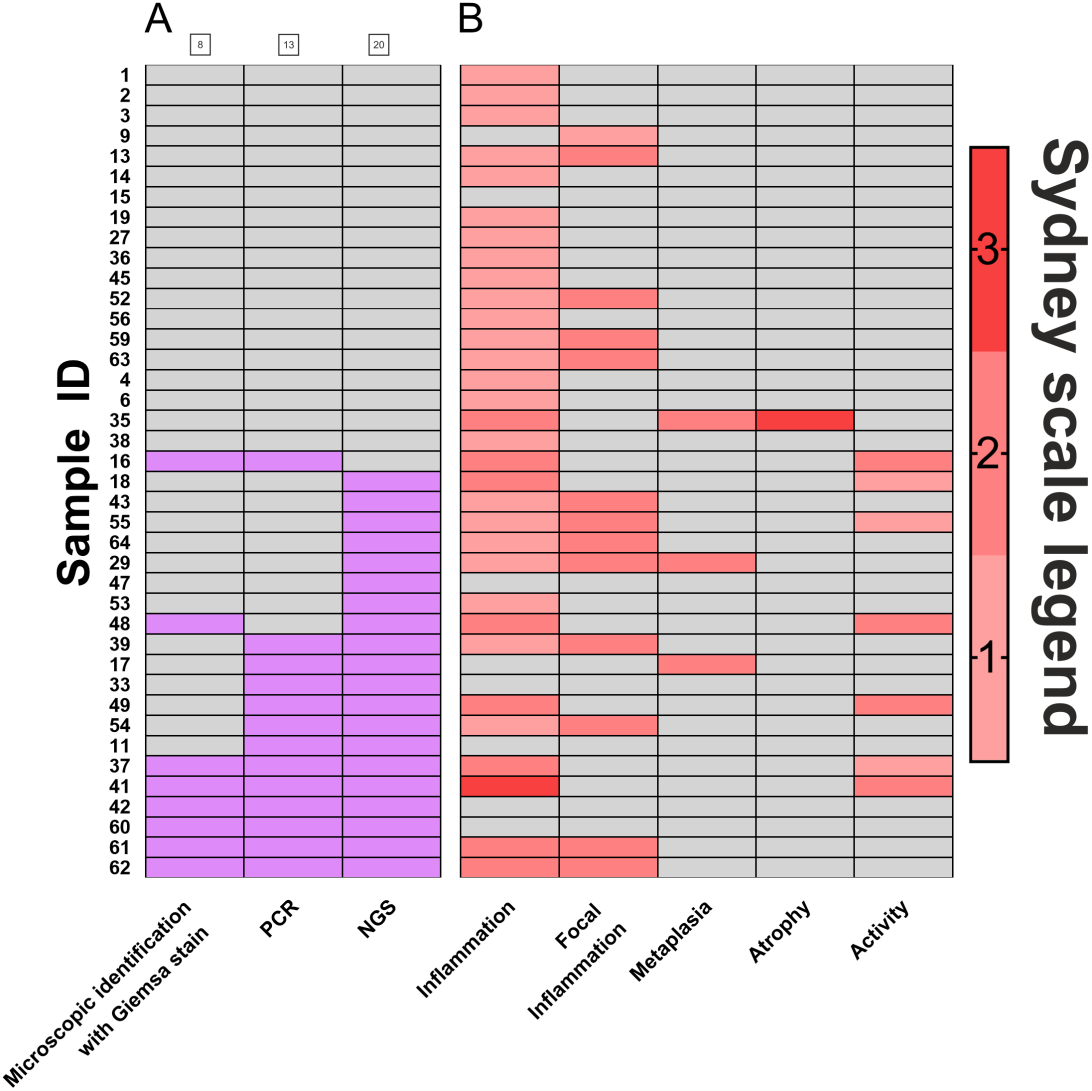
Comparison of *H. pylori.* detection methods. (A) Frequency of *Helicobacter pylori* detection in the same patients by three different detection methods: microscopic identification with Giemsa stain (according to Sydney Scale), PCR detection of urease gene in bioptates, 16S rRNA sequencing in bioptates, grey- *H. pylori* not detected in the sample (negative), red- *H. pylori* detected in the sample (positive); (B) assessment by Sydney System parameters (0–3). Numbers of individual samples are in accordance to relevant numbers of samples in Biobank of RSH and to raw data uploaded with this manuscript as Supplemental Information.

Biopsies of the antral part of the stomach (*N* = 40) were used to compare the efficacy of *H. pylori* detection by three diagnostic methods: histopathological assessment and scoring according to Sydney classification system (standard diagnostics of patients, based on eosin, hematoxylin and Giemsa stain), PCR detection of urease (ureA), and detection by the sequencing of regions coding for 16S rRNA. Results of this comparison are presented in Fig. 1. Histological diagnostics with Giemsa staining revealed *H. pylori* infection in 20% (8 out of 40) of patients. Molecular biology technique based on PCR allowed for *H. pylori* detection in 32.5% (13 out of 40) of patients. 16s rRNA sequencing, however, demonstrated *H. pylori* presence in 50% (20 out of 40) of examined patients. Thus, in 17.5% of infections were identified only by this method. McNemar’s test was used to compare statistically significant differences between these three diagnostic approaches, and results were fitted into 2x2 tables as Positive-Positive, Positive-Negative and Negative-Negative cases (Table 1). No statistically significant differences between microscopic identification with Giemsa stain and PCR detection was identified. However, 16S rRNA sequencing significantly differed in its sensitivity from both microscopic identification (*p* < 0.002) and PCR detection (*p* < 0.05).

**Table 1:** Comparison of *H. pylori* detection rates. (A) 16S rRNA sequencing v. Giemsa staining, (B) 16S rRNA sequencing v. PCR, and (C) PCR v. Giemsa staining; Positive, number of samples identified as positive for *H. pylori*; Negative, number of samples identified as negative for *H. pylori*.

**A**		16S rRNA sequencing	
		Positive	Negative
Giemsa staining during histological assessment	Positive	7	1
	Negative	13	19

**B**		16S rRNA sequencing	
		Positive	Negative
PCR	Positive	12	1
	Negative	8	19

**C**		PCR	
		Positive	Negative
Giemsa staining during histological assessment	Positive	7	1
	Negative	6	26

Further, analysis of scores assigned by the Sydney system (Fig. 1B) revealed that polymorph activity (neutrophilic infiltration) significantly correlated to the detection of *H. pylori* by 16S rRNA sequencing (*p* < 0.01) while no significant correlation was observed for microscopic identification with Giemsa stain or for PCR detection. No correlations for possible metaplasia, inflammation, or atrophy were found (Fig. 1B, Table S1).

## Discussion

Comparison of three techniques of *H. pylori* detection showed that microbiome diagnostics by NGS with 16S rRNA sequencing offered the most sensitive method for *H. pylori* identification. According to the NGS-PCR-histopathology comparison presented herein, the 16S rRNA sequencing identified *H. pylori* infections in 17,5% more patients than other investigated methods. Moreover, only 16S rRNA sequencing was significantly correlated to the important evaluation by Sydney system scoring of polymorph activity. In one case *H. pylori* was detected by NGS and histology, but PCR yielded a negative result. Only in one case histology- and PCR-based testing yielded positive results while NGS-based 16S rRNA sequencing did not detect *H. pylori.* Microscopic assessment with Giemsa staining of histological samples seems to be the least sensitive method and it did not allow *H. pylori* to be detected in 12 out of 20 patients identified as *Helicobacter pylori*-positive by 16S rRNA sequencing. Statistical analysis demonstrated that 16S rRNA test significantly more frequently reported positive detection of *H. pylori* than two other investigated methods (Table 1). Notably, PCR seemed to be considered so far as the most sensitive (Ramírez-Lázaro et al., 2011; Chen et al., 2012; Kiss et al., 2016; Mnena, Emmanuel & Rai, 2017). Importantly, in this study we used exactly the same DNA samples for both PCR and NGS reactions, and we got significantly more positive identifications by NGS. This may be due to the differences in the genetic regions targeted in both reactions: in PCR this is typically the urease coding gene (*ureA*), while 16S rRNA is coded in a different part of bacterial genome (He et al., 2002; Patel et al., 2014a; Samareh-Fekri et al., 2016). Thus, they may differ in their sequence and related physical properties, accessibility, and thus they may differ in their detection rate. Also, NGS procedure may allow for reducing the amount of impurities that potentially limit PCR detection, as well as the high sensitivity of NGS adds to the detection of a low represented bacterial strain. In one case (patient no. 35) characteristic symptoms were observed: inflammation, metaplasia, atrophy, however no *H. pylori* presence detected by any applied detection method. We suppose that other (than *H. pylori*) factors accounted to these pathological symptoms. It has been demonstrated, that e.g., environmental factors including chemical compounds, or improper pharmacological treatment may induce pathological changes in stomach. Nevertheless, in this case no specific identification of the causative factor was available.

In spite of the demonstrated high sensitivity, there are potential limitations for NGS-based detection. First, the difficulty in discriminating between active, or at least living (coccoid), bacteria from killed ones (e.g., by antibiotics). The same limitation applies to other methods based on bacterial DNA detection, including PCR. However, specific conditions in the stomach, including low pH, enzymes and peristaltic movements, strongly limit the possibility for inactivated microorganisms or their debris to persist in the stomach. Thus, long delays in *H. pylori* detection after its inactivation seems unlikely. The second limitation for a rapid, wide application of 16S rRNA sequencing by NGS as a routine diagnostic tool is the necessity of specialized equipment and compounds for sample processing and analysis, as well as still relatively high costs and time required. Nevertheless, recent development of the NGS-based methods suggest their prospective wider application in the future. Costs and time consuming characteristics of methods used in this study are presented in Table 2. As the more sensitive, this method might contribute to better understanding *of H. pylori* epidemiology. Prevalence of *H. pylori* in humans is widely studied around the world including meta-analyses of diagnostic data. Most infected people are identified in Africa –on average 70% individuals are hosts for that bacterial species. Oceania has the lowest rate of *H. pylori* prevalence –24.4%. Europe should be analysed by regions, e.g., according to global reports in Eastern Europe nearly 64% of people are infected by *H. pylori.* That value is above the European average, which is 49.18% (Hooi et al., 2017). All these estimations are based on *H. pylori* detection by one of the following: *H. pylori* serology, stool antigen detection, urea breath test, biopsies for *Campylobacter*-like organism test, rapid urease test, histology and culture. One should bear in mind that these methods may lead to underestimation; thus the overall frequency of *H. pylori* in humans could be identified as higher provided highly sensitive NGS methods are used.

**Table 2:** Comparison of time and costs required in *H. pylori* detection by 16S rRNA sequencing, PCR, and Giemsa staining. Each row represents method used in this study with its features related to time needed to receive results and approximate costs.

Method	Time	Costs
16S rRNA gene sequencing	• Results available in 24 h • Initial library preparation required	• Cost highly variable, from very high to significantly decreased if big number of samples are analysed in parallel • Special instruments are needed
Polymerase Chain Reaction	• Results in a few hours	• Relatively low cost, the key is DNA isolation method • standard thermocyclers are sufficient
Giemsa staining during histological assessment	• Typically within days	• No special instruments needed • Low cost reagents

*Helicobacter pylori* is one of the most important factors inducing gastrointestinal diseases. Methods for its diagnostics and prevention are constantly being developed. The frequency of *H. pylori* infections and demonstrated high sensitivity of molecular methods strongly support reconsidering standards of diagnostics of *H. pylori* infections. Here we have demonstrated that novel methods of identification of human microbiome components are fully applicable and even more sensitive than standard methods for detection of *H. pylori*. 16S rRNA sequencing by NGS can be employed for identification of infections caused by this bacterium.

## Conclusions

16S rRNA sequencing allowed for detection of *H. pylori* infection in 20% more patients than histology- and PCR-based methods. Our study showed that *H. pylori* detection by 16S rRNA sequencing correlated to polymorph activity revealed by Sydney scoring. It should be considered in the future to extend current routine diagnostics of *H. pylori* infections with NGS applications.

##  Supplemental Information

10.7717/peerj.9099/supp-1 Supplemental Information 1 Data used in correlation verification Results presented in the table were used in correlation occurence using McNemar test.

10.7717/peerj.9099/supp-2 Supplemental Information 2 Electrophoresis 1/4 Samples 1-16, agarose 2% with EtBr at 100 ms. Visible ureA PCR product.

10.7717/peerj.9099/supp-3 Supplemental Information 3 Electrophoresis 2/4 Samples 17-39, agarose 2% with EtBr at 100 ms. Visible ureA PCR product.

10.7717/peerj.9099/supp-4 Supplemental Information 4 Electrophoresis gel 3/4 Samples 41-55, agarose 2% with EtBr at 100 ms. Visible ureA PCR product.

10.7717/peerj.9099/supp-5 Supplemental Information 5 Samples Samples 56-64, agarose 2% with EtBr at 100 ms. Visible ureA PCR product.

10.7717/peerj.9099/supp-6 Supplemental Information 6 Electrophoresis Gel analyzed in Fiji 1/4 Each part of image contains plot lanes of electrophoresis gel in H. pylori PCR detection**

10.7717/peerj.9099/supp-7 Supplemental Information 7 Electrophoresis Gel analyzed in Fiji 2/4 Each part of image contains plot lanes of electrophoresis gel in H. pylori PCR detection

10.7717/peerj.9099/supp-8 Supplemental Information 8 Electrophoresis Gel analyzed in Fiji 3/4 Each part of image contains plot lanes of electrophoresis gel in H. pylori PCR detection

10.7717/peerj.9099/supp-9 Supplemental Information 9 Electrophoresis Gel analyzed in Fiji 4/4 Each part of image contains plot lanes of electrophoresis gel in H. pylori PCR detection

10.7717/peerj.9099/supp-10 Supplemental Information 10 Histology results Results prepared by diagnostician according to Sydney Scale

## Additional Information and Declarations

### Data Availability

The following information was supplied regarding data availability: The data analysed in this experiment is available online on MG-RAST (MG-RAST ID: mgp86818).
https://www.mg-rast.org/linkin.cgi?project=mgp86818.
