# Supplementary figures and images for: Application of 16S rRNA gene sequencing in Helicobacter pylori detection
Source: PeerJ. 2020 May 13;8:e9099. doi: 10.7717/peerj.9099 (PMC7229771; doi:10.7717/peerj.9099)

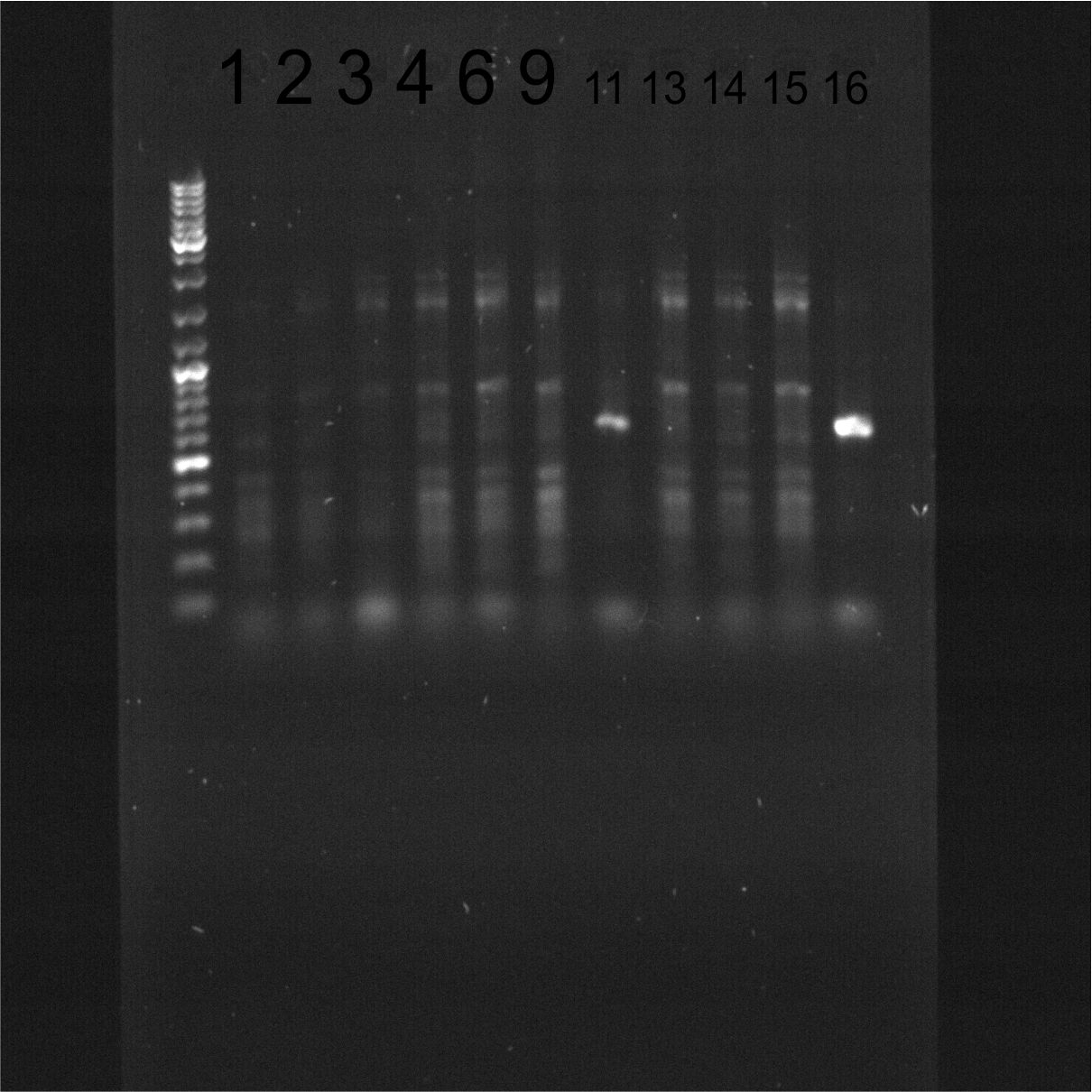

Supplement: Supplemental Information 2 — Samples 1-16, agarose 2% with EtBr at 100 ms. Visible ureA PCR product. [file peerj-08-9099-s002.jpg]

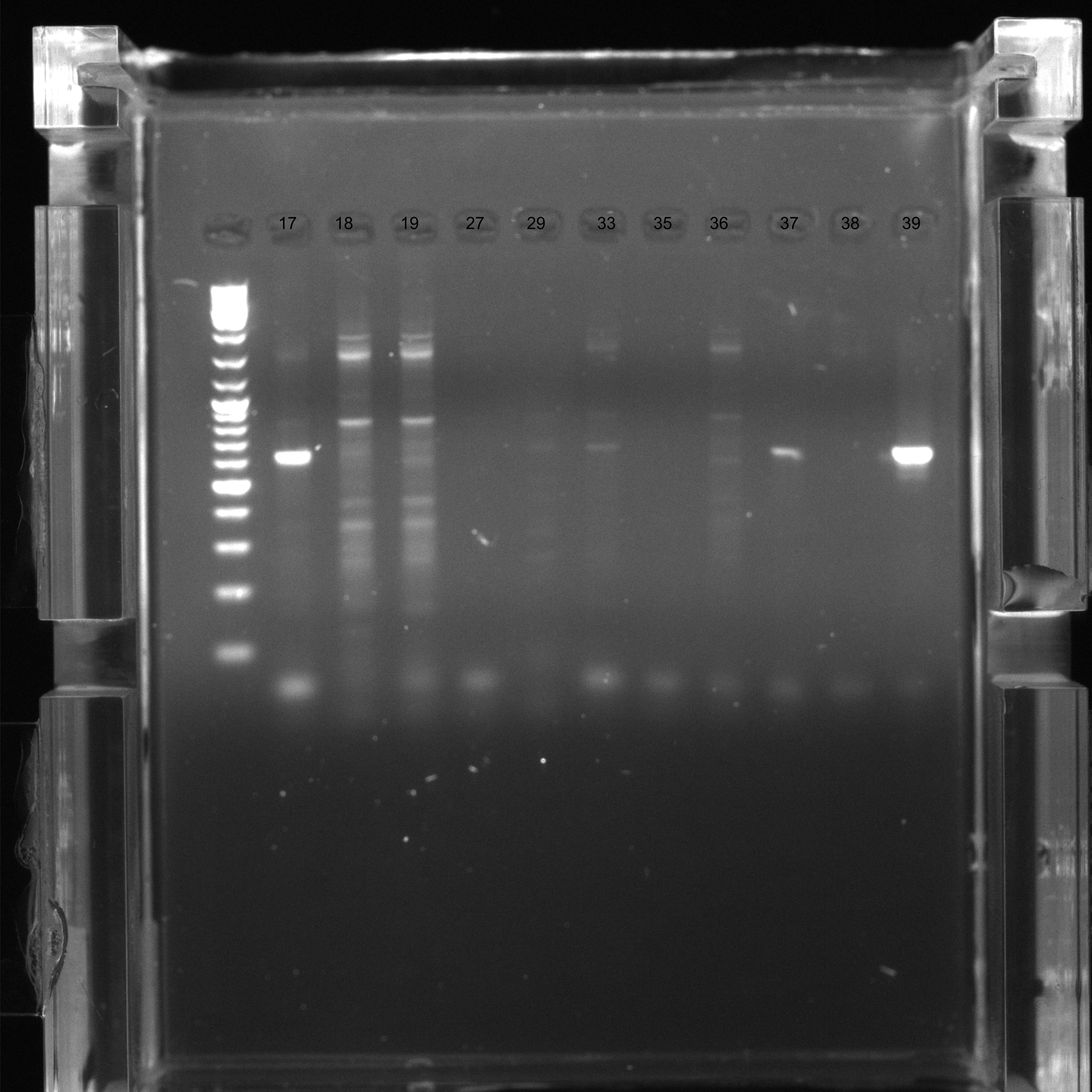

Supplement: Supplemental Information 3 — Samples 17-39, agarose 2% with EtBr at 100 ms. Visible ureA PCR product. [file peerj-08-9099-s003.jpg]

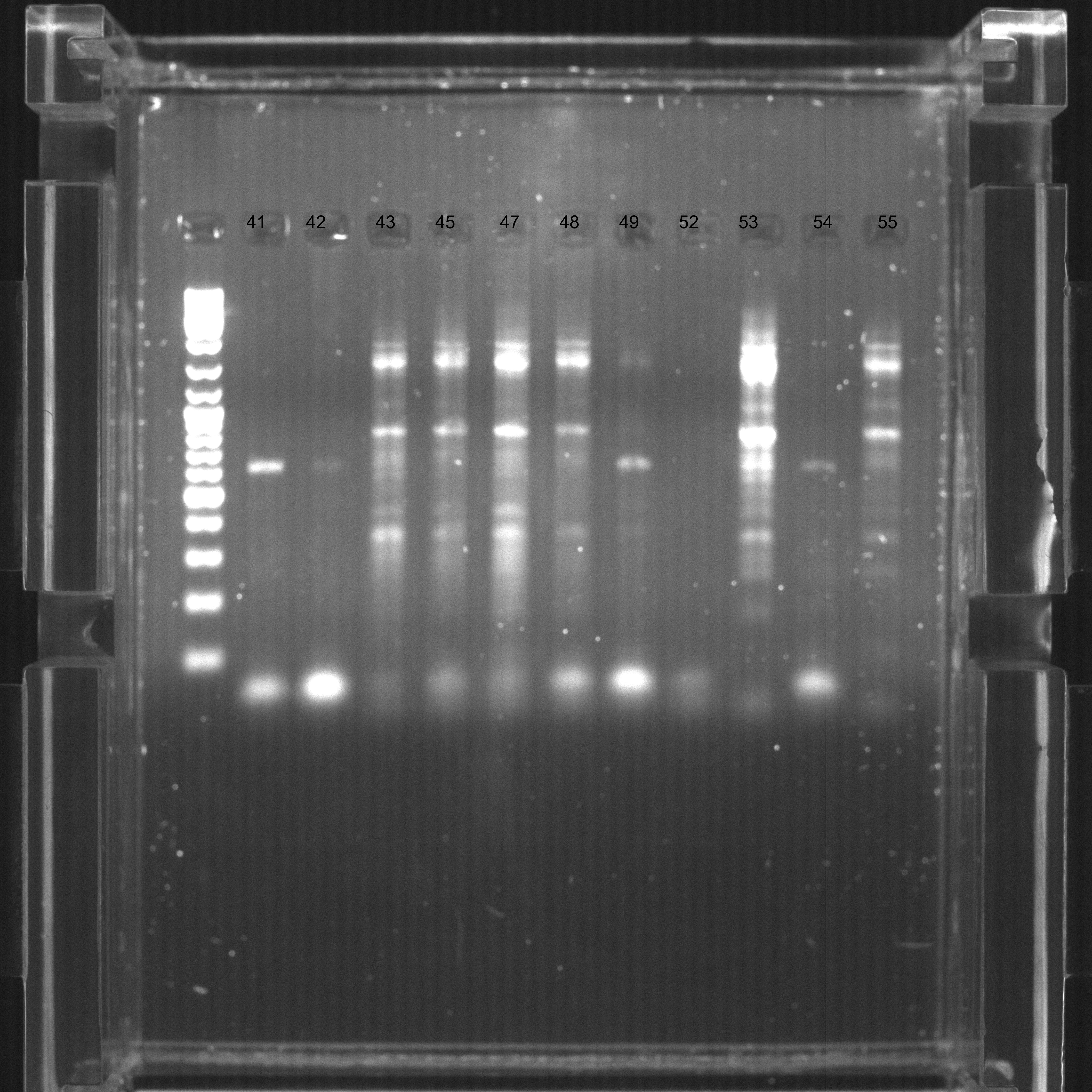

Supplement: Supplemental Information 4 — Samples 41-55, agarose 2% with EtBr at 100 ms. Visible ureA PCR product. [file peerj-08-9099-s004.jpg]

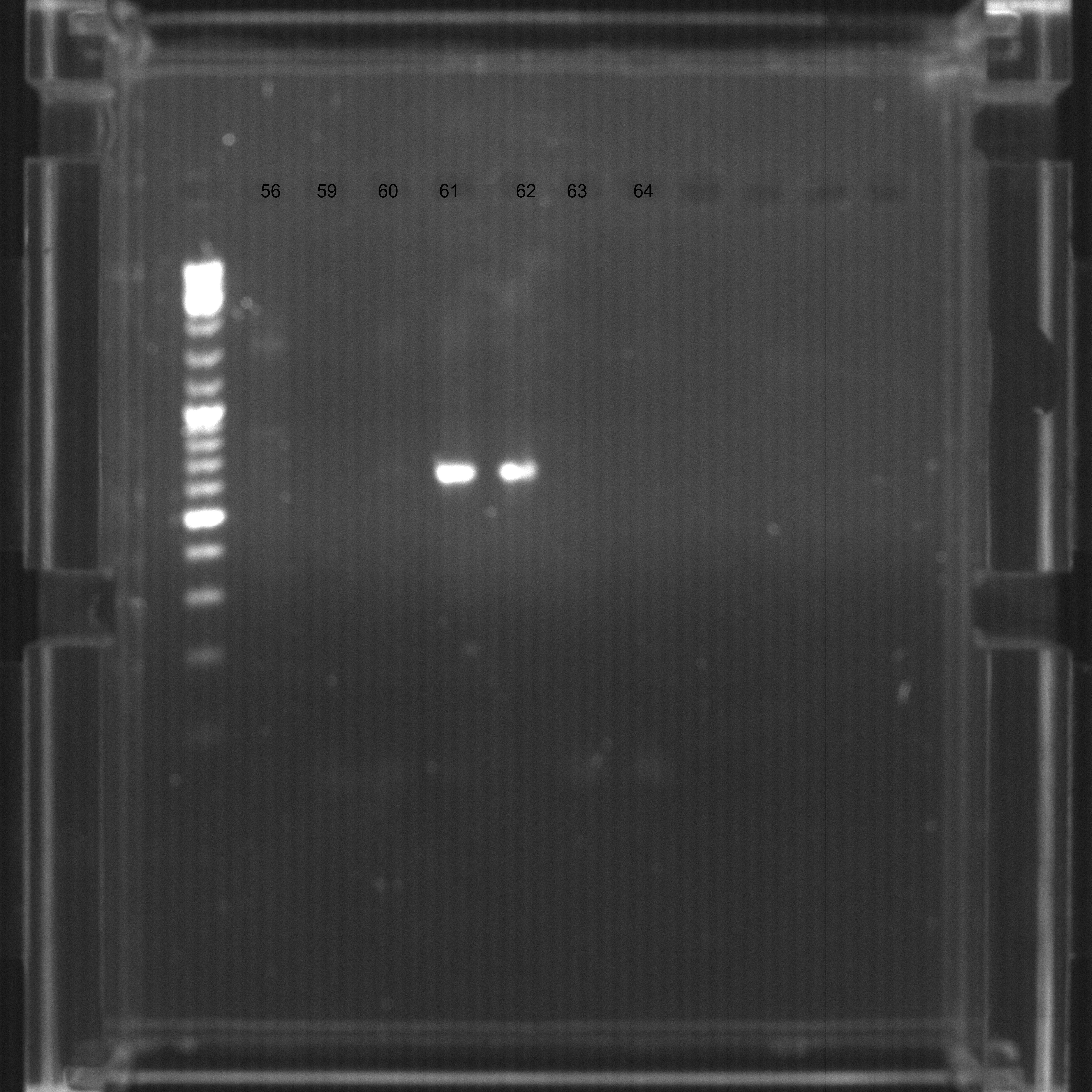

Supplement: Supplemental Information 5 — Samples 56-64, agarose 2% with EtBr at 100 ms. Visible ureA PCR product. [file peerj-08-9099-s005.jpg]

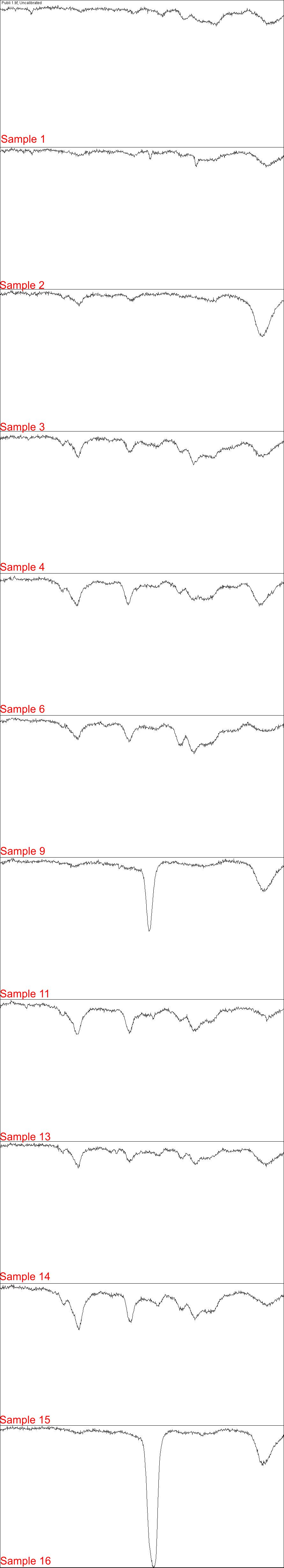

Supplement: Supplemental Information 6 — Each part of image contains plot lanes of electrophoresis gel in H. pylori PCR detection [file peerj-08-9099-s006.jpg]

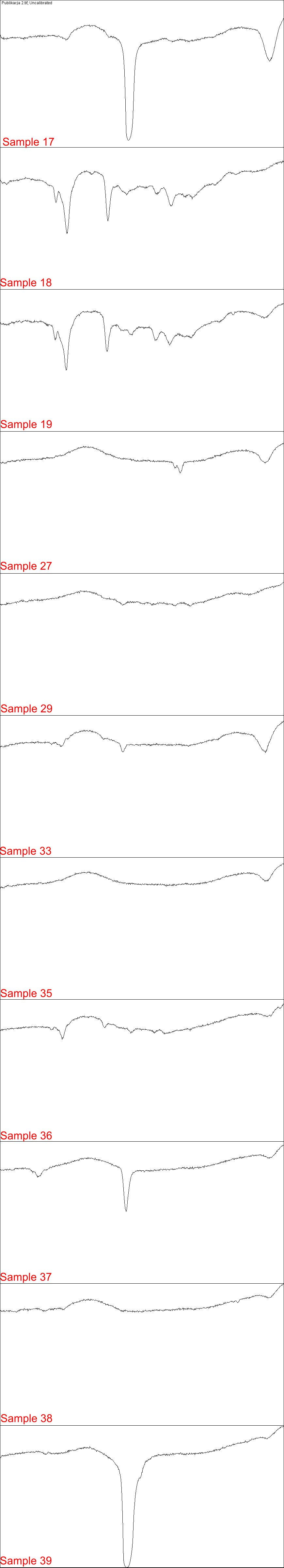

Supplement: Supplemental Information 7 — Each part of image contains plot lanes of electrophoresis gel in H. pylori PCR detection [file peerj-08-9099-s007.jpg]

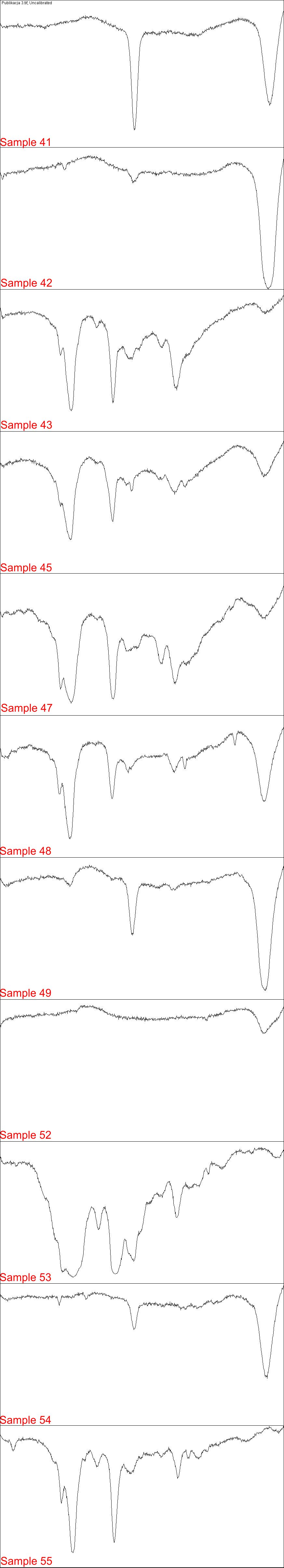

Supplement: Supplemental Information 8 — Each part of image contains plot lanes of electrophoresis gel in H. pylori PCR detection [file peerj-08-9099-s008.jpg]

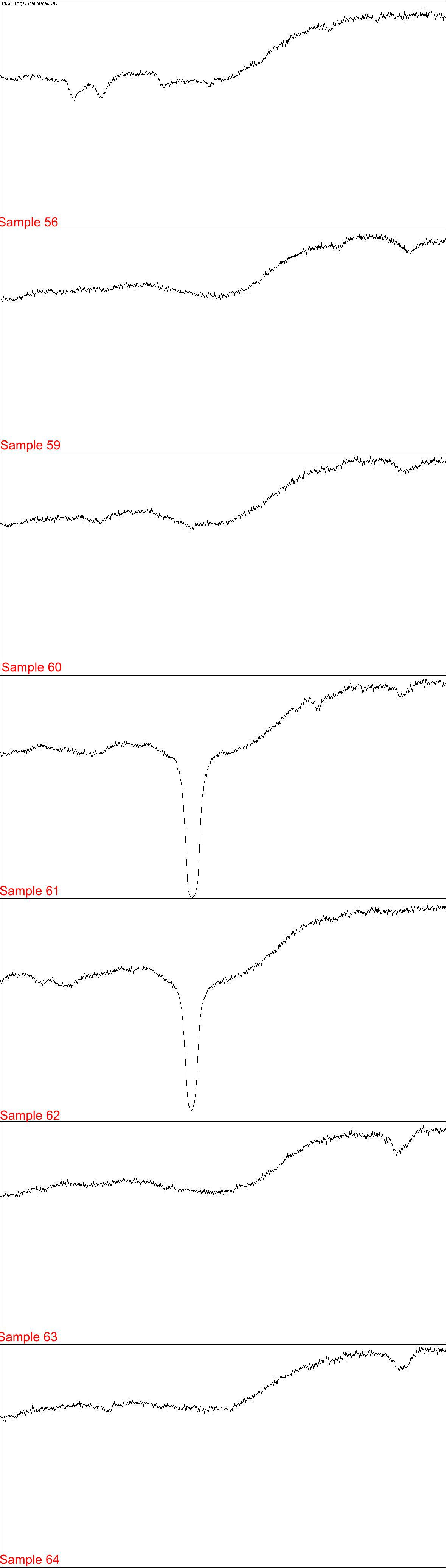

Supplement: Supplemental Information 9 — Each part of image contains plot lanes of electrophoresis gel in H. pylori PCR detection [file peerj-08-9099-s009.jpg]
